# A Glycyrrhetinic Acid-Modified Curcumin Supramolecular Hydrogel for liver tumor targeting therapy

**DOI:** 10.1038/srep44210

**Published:** 2017-03-10

**Authors:** Guoqin Chen, Jinliang Li, Yanbin Cai, Jie Zhan, Jie Gao, Mingcai Song, Yang Shi, Zhimou Yang

**Affiliations:** 1 Cardiology Department of Panyu Central Hospital, Guangzhou, China; Cardiovascular Disease Institute of Panyu District, Guangzhou, Guangdong 511400, P. R. China; 2 Guangzhou University of Chinese Medicine, Guangzhou, Guangdong 510006, Cardiology Department of Panyu Central Hospital, Guangzhou, Guangdong 511400, P. R. China; 3 Key Laboratory of Bioactive Materials, Ministry of Education, College of Life Sciences, Nankai University, Tianjin 300071, P. R. China; 4 State Key Laboratory of Medicinal Chemical Biology, Nankai University, Tianjin 300071, P. R. China

## Abstract

Curcumin (Cur), a phenolic anti-oxidant compound obtained from Curcuma longa plant, possesses a variety of therapeutic properties. However, it is suffered from its low water solubility and low bioavailability property, which seriously restricts its clinical application. In this study, we developed a glycyrrhetinic acid (GA) modified curcumin supramolecular pro-gelator (GA-Cur) and a control compound Nap-Cur by replacing GA with the naphthylacetic acid (Nap). Both compounds showed good water solubility and could form supramolecular gels by disulfide bond reduction triggered by glutathione (GSH) *in vitro*. Both formed gels could sustainedly release Cur in buffer solutions. We also investigated the cytotoxicity of pro-gelators to HepG2 cells by a MTT assay and determined the cellular uptake behaviours of them by fluorescence microscopy and LC-MS. Due to the over expression of GA receptor in liver cancer cells, our pro-gelator of GA-Cur showed an enhanced cellular uptake and better inhibition capacity to liver tumor cells than Nap-Cur. Therefore, the GA-Cur could significantly inhibit HepG2 cell growth. Our study provides a novel nanomaterial for liver tumor chemotherapy.

Hepatocellular carcinoma (HCC) is one of the most common cancers in the world with a high mortality rate and few effective treatment options^1^,^2^. Surgery in combine with chemotherapy are usually the necessary and to date the main treatments for HCC. However, current chemotherapeutic agents have several shortcomings including low aqueous solubility, poor tumor selectivity and considerable side-effects to normal tissues^3^,^4^. Thus, it is very important to investigate and develop novel drug delivery systems to selectively deliver chemotherapeutic agents to liver cancer. Actually, over the past few decades, targeted drug delivery systems, especially active targeting, have shown enormous potential in cancer therapy because of their improved specificity and efficacy of anti-carcinogen and the minimized side effects^5^,^6^.

Glycyrrhetinic acid (GA), a pentacyclic triterpene acid, is the main bioactive compound extracted from the herb liquorice. As a targeting ligand, GA has been demonstrated to specifically bind to liver cell membrane, because of the abundant GA receptors on hepatocyte membranes^7^,^8^. Besides, the GA receptors, identified as protein kinase C, are significantly over-expressed in HCC cells than other normal cells^9^,^10^. Therefore, GA has been widely used as a targeting ligand to functionalize nanomaterials to treat HCC, including micelles^7^,^11^,^12^, liposomes^8^,^9^,^13^, and nanospheres^14^,^15^,^16^,^17^. As results, these GA modified nanomaterials exhibited enhanced uptakes by hepatocyte and HCC cells and better inhibition effects to HCC than unmodified ones.

Curcumin (Cur), a hydrophobic polyphenol compound, is derived from the rhizomes of Curcuma longa. Cur has a wide range of therapeutic properties, such as anti-inflammation^18^, anti-oxidant^19^, anti-mutagenic^20^, and anticancer^21^ with low or no intrinsic toxicity to healthy cells. Moreover, emerging evidences show that Cur can prevent and suppress the generation, transformation, proliferation and metastasis of many types of cancer cells including breast cancer, colon carcinoma, cervical cancer, stomach cancer, pancreatic cancer, liver cancer cells^22^,^23^. Furthermore, Cur shows great promise as a chemo-preventive and therapeutic drug in HCC, possibly because of its potent anti-angiogenic activity and pro-apoptotic properties in human HepG2 cells^24^. However, the clinical application of Cur remains very limited due to its extremely poor aqueous solubility (≈11 ng/ml)^25^ and low bioavailability^26^. Various approaches have been attempted to address the aforementioned problems of Cur including using biomaterials such as the polymeric micelles^27^,^28^,^29^,^30^, liposomes^31^,^32^,^33^, polymeric nanoparticles (NPs)^34^,^35^,^36^, and hydrogels^37^,^38^,^39^ to deliver Cur.

As a promising biomaterial and drug carrier, supramolecular hydrogels based on short peptide and therapeutic agents have attracted extensive research interests because of the high drug loadings, sustained and responsive drug release property, good biocompatibility, ease of design and synthesis, etc^40^,^41^,^42^. Several examples of supramolecular hydrogel of therapeutic agents including Olsalazine^43^, Taxol^44^,^45^,^46^, Naprofen^47^, Camptothecin^48^, and Curcumin^39^ have been rationally synthesized and reported. However, examples of *in situ* formed supramolecular hydrogels with targeting effects to cancer cells are rare. Here we designed and synthesized a glycyrrhetinic acid (GA) modified curcumin supramolecular pro-gelator (GA-Cur) with a targeting effect to liver cancer cells, and we also demonstrated its improved cellular uptake and better inhibition capacity to HepG2 cells than a control compound without the targeting effect.

## Results

### Molecular design and synthesis

We recently observed that a pro-gelator of Cur-FFE-ss-ERGD could inhibit cancer cell growth more efficiently than the corresponding gelator of Cur-FFE-s because of the enhanced cellular uptake and evenly cellular distribution of the pro-gelator^39^. We also demonstrated that a pro-gelator of taxol (Taxol-FFpY) exhibited a better inhibition effect to cancer cells than the corresponding gelator of Taxol-FFY because of the similar mechanism^45^. Xu and Liang groups also demonstrated that the intracellular formation of nanofibers or nanoparticles could significantly inhibit taxol-resistant cancer cells growth^46^,^49^. These results suggested that pro-gelators capable of forming nanomedicines within cells by intracellular catalysts might possess better inhibition capacities to cancer cells. Based on these pioneering works, we imaged that, by integrating a targeting ligand to the pro-gelator, the inhibition capacity of pro-gelators to cancer cells might be further improved.

**Figure 1:**
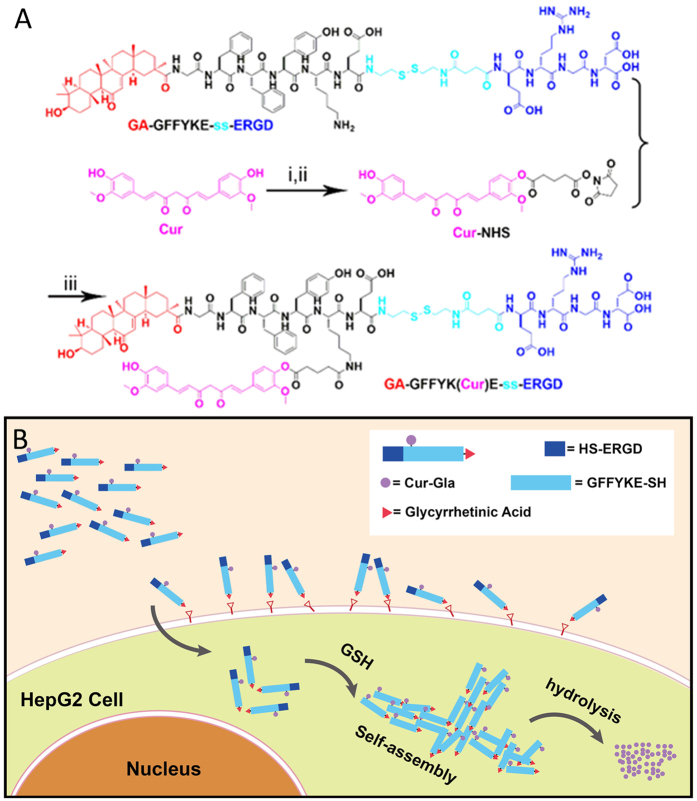
(**A**) Chemical structures and synthetic route for the GA–GFFYK(Cur)E-ss-ERGD (i: glutaricacid anhydride, pyridine, ii: N-hydroxy succinimide (NHS), dicyclohexylcarbodiimide (DCC), DMF, iii: DMF, DIPEA), (**B**) diagram to illustrate the mechanism of our compounds for tumor targeting therapy.

These pioneering results and our hypothesis stimulated us to design a pro-gelator GA–GFFYK(Cur)E-ss-ERGD (GA-Cur in Fig. 1A) with a targeting ligand of GA to liver cancer cells. The pro-gelator might be converted to GA–GFFYK(Cur)E-s (GA-gelator) by disulfide bond reduction triggered by glutathione (GSH). We believed that, with the assistance of targeting ligand of GA, the pro-gelator could show a better selectivity to liver cancer cells than normal cells (Fig. 1B). As shown in Fig. 1A, we firstly synthesized the peptide derivate of GA-GFFYKE-ss-ERGD by standard Fmoc-solid phase peptide synthesis (SPPS), which was then used to couple with N-hydroxysuccinimide (NHS)-activated Curcumin Glutaric acid (Cur-Gla). The pure compound GA-Cur was obtained by reverse phase high performance liquid chromatography (HPLC) with a moderate yield (about 20%). We also replaced the targeting ligand of GA with naphthalene acetic acid (Nap) to make a control compound of Nap–GFFYK(Cur)E-ss-ERGD (Nap-Cur)using the similar synthetic route.

### Gelation test and characterization of hydrogels

**Figure 2:**
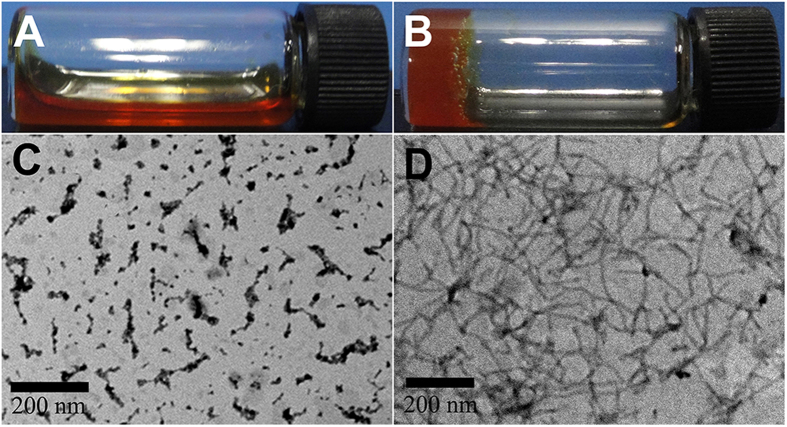
Optical images of (**A**) PBS solutions containing 1 wt% of the precursors (GA-Cur) and (**B**) the hydrogel (GA-gel) formed by treating solution in (**A**) with 4 equiv. of GSH, (**C**) transmission electron microscopy (TEM) image of the precursors (GA-cur) and (**D**) transmission electron microscopy (TEM) image of the hydrogel (GA-gel).

We therefore tested the gelation ability of the obtained two compounds. The pro-gelator GA-Cur and Nap-Cur could be well solubilized in phosphate buffer saline (PBS, pH = 7.4) at a concentration of 10 mg/mL (1 wt%, Fig. 2A; Fig. S-6A). The critical micelle concentration (CMC) of them was about 1105 and 1349 μg/mL (Fig. S-7), respectively, which were much higher than the aqueous solubility of curcumin (11ng/mL). After adding 4 equiv. of GSH to the solution, yellowish supramolecular hydrogels (GA-gel, Fig. 2B; Nap-gel, Fig. S-6B) could be obtained after about 1.5 h at 25 °C. The minimum gelation concentration of GA-Cur was about 0.75 wt%. The LC-MS traces clearly indicated that the pro-gelator GA-cur was converted by GSH to the gelator of GA–GFFYK(Cur)E-s (Fig. S-8). Similar gelation property was observed for Nap-cur (Fig. S-9). The hydrogels were stable and would not change the appearance for more than a month at room temperature. We then used a rheometer to characterize the resulting hydrogels obtained at 2 h time point. As shown in Fig. S-10, the value of elasticity (G′) for the GA-gel was about 150 Pa, and that of viscosity (G″) was about 12 Pa. The value of elasticity (G′) and viscosity (G″) for the Nap-gel was 1000 and 100 Pa, respectively, suggesting a better mechanical property of Nap-gel than GA-gel. We then investigated the fluorescence spectra of the precursors (GA-cur and Nap-cur) and the hydrogels (GA-gel and Nap-gel). As shown in Fig. S-11, with the amount of GSH increased, the fluorescence of GA-cur and Nap-cur quenched gradually, which was due to well-known aggregation caused quenching phenomena.

### Release profile and *In vitro* inhibition capacity

**Figure 3:**
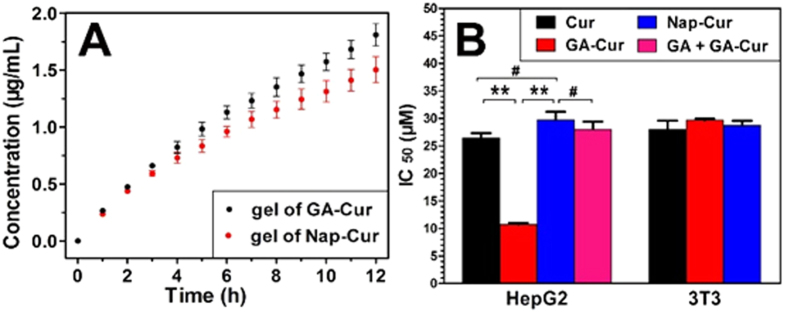
(**A**) Accumulative release profile at 37 °C in PBS buffers (pH = 7.4) of Cur from GA-gel and Nap-gel, respectively and (**B**) Cytotoxicity of Cur, GA-Cur, and Nap-Cur against HepG2 and mouse NIH 3T3 cells (the IC_50_ of GA + GA-Cur was evaluated against HepG2 cells by pre-treating cells with 2 equiv. of free GA to block the GA receptors for 2 hours and then replacing with GA-Cur for another 48 hours, ^#^P > 0.5, **P < 0.01).

Cur could be released from gels by ester bond hydrolysis. We then determined the release profile of Cur from both gels. As shown in Fig. 3A, both Nap-gel and GA-gel could sustainedly release Cur during the 12 h experimental period, and the release speed of Cur was about 1.5 and 1.85 μg/mL at 37 °C from GA-gel and Nap-gel, respectively. We next evaluated the inhibition capacity of the Cur, GA-Cur, Nap-Cur to both HepG2 and 3T3 cells. After incubating the cells with different compounds at a series of concentrations for 48 h, an MTT assay was performed. As shown in Fig. 3B, the Cur, GA-Cur, Nap-Cur exhibited an IC_50_value of 26.5, 10.7, and 29.7 μM against HepG2, respectively. For mouse fibroblast 3T3 cells, compounds of Cur, GA-Cur, and Nap-Cur showed similar IC_50_ values, which was 28.0, 29.6 and 28.7 μM, respectively.

### Confocal microscopy and cellular uptake

**Figure 4:**
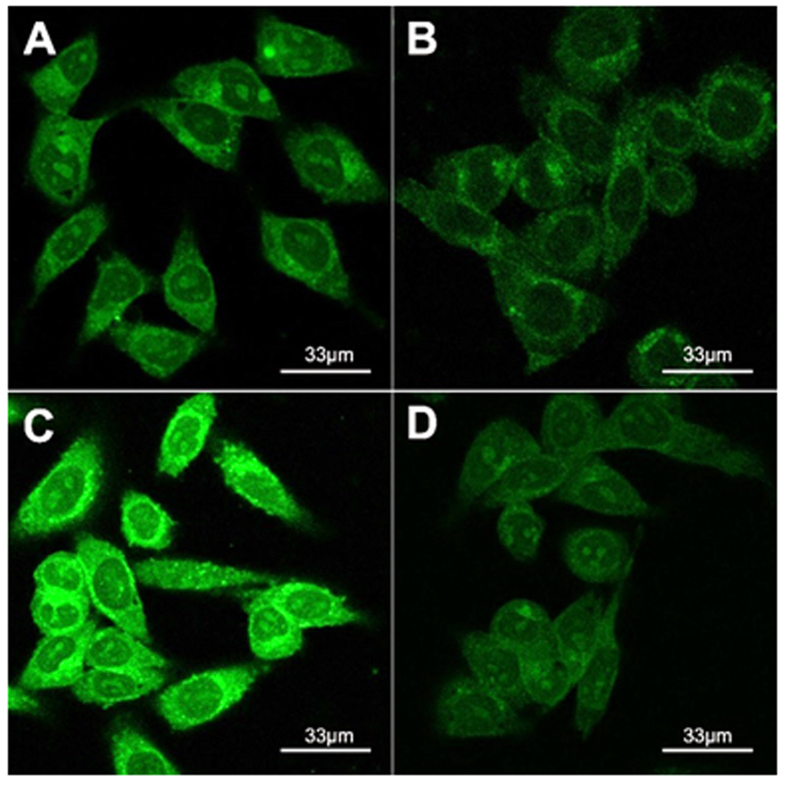
Confocal fluorescence microscopy images of HepG2 cells treated with25 μM of (**A**) Cur, (**B**) Nap-Cur, (**C**) GA-Cur, and (**D**) GA + GA-Cur for 4 hours.

**Figure 5:**
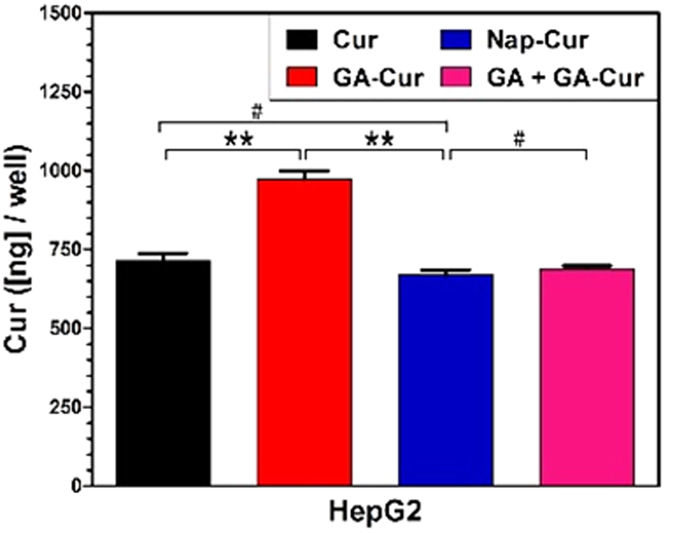
The amount of Curcumin in HepG2 cells treated with different compounds (^#^P > 0.5, **P < 0.01).

In order to understand the best inhibition capacity of GA-Cur, we obtained confocal fluorescence microscopy images of HepG2 cells treated with Cur, GA-Cur, Nap-Cur and GA + GA-Cur for 4 h. As shown in Fig. 4, we observed green fluorescence from the cytoplasm of the cells treated with different compounds. However, cells treated with GA-Cur showed the strongest green fluorescence (Fig. 4C), compared with those treated with Cur (Fig. 4A) and Nap-Cur (Fig. 4B) at the same concentration. If we firstly treating HepG2 cells with 2 equiv. of GA for 2 hand then with GA-Cur for another 4 h, the cells exhibited similar intensity of green fluorescence (Fig. 4D) to those treated with Cur and Nap-Cur. When we treated the HepG2 cellswith GA-cur and inhibitors (GA) together, we found that the green fluorescence of cells were also stronger than that of GA + GA-cur (Fig. S-12). These results indicated that GA-cur showed good targeting effect on HepG2 cells. We also determined the intra-cellular concentration of compounds. HepG2 cells were incubated with Cur, GA-Cur, Nap-Cur, or GA + GA-Cur (2:1) for 4 h, and then they were collected to determine the intracellular concentration of Cur. The results in Fig. 5 indicated that the intra-cellular concentration of Cur was 715, 973, 670 and 689 ng/well for Cur, GA-Cur, Nap-Cur, or GA + GA-Cur, respectively. In order to investigate whether the hydrogels have the similar inhibition capacity as pro-gelators, we took confocal fluorescence microscopy images of HepG2 cells treated with GA-gel and Nap-gel at 4 h time point containing 25 μM curcumin. As shown in Fig. S-13, an extremely weak green fluorescence was observed in HepG2 cells. The results indicated that the cellular uptake of hydrogels were less than that of pro-gelators, which was consistent with our previous results that gels would show much lower cellular uptake than the pro-gelator^50^.

## Discussion

According to the rheology results (Fig. S-10), both the G′ and G″ of the gel showed weak frequency dependences at the frequency range from 0.1 to 100 rad/s, and the G′ value of the gel was more than 10 times bigger than its corresponding G″ value, suggesting the formation of a true gel. We also obtained a transmission electron microscopy (TEM) image to characterize the self-assembled nanostructures in the gel. As shown in Fig. 2C, the pro-gelator of GA-cur showed irregular short fibers in solutions, while the GA-gel exhibited uniform nanofibers with diameters of about 10 nm (Fig. 2D). The flexible nanofibers entangled with each other to form three dimensional (3D) networks for hydrogel formation. Similar to GA-Cur, the control compound Nap-Cur also formed a supramolecular hydrogel (Nap-gel) with nanofibers in the gel (Fig. S-6).

The smaller IC_50_ value (better inhibition capacity) of GA-Cur than that of Nap-Cur suggested the targeting effect of GA to HepG2 cells. If firstly treating the HepG2 cells with GA for 2 h and then with GA-Cur for another 4 h, the GA + GA-Cur group exhibited an IC_50_value of 28 μM, which further demonstrated the targeting effect of GA to HepG2 cells. The results of IC_50_ value and cellular uptake hadclearly indicated that GA helped the accumulation of GA-Cur in HepG2 cells possibly due to the specific ligand-receptor interaction between GA and GA receptor. In order to further prove this conclusion, we performed control experiments in 3T3 cells. As shown in Fig. S-14, the fluorescent intensity of cells treated with Cur, GA-Cur, Nap-Cur, or GA + GA-Cur was similar. The intra-cellular concentration of Cur for them was also similar, which was 667, 681, 641 and 626 ng/well, respectively (Fig. S-15). These observations further demonstrated the targeting effect of GA to HepG2 cells.

### Summary

In summary, we have developed a glycyrrhetinic acid-modified curcumin supramolecular pro-gelator (GA-Cur), which could be converted to a supramolecular hydrogelator and form a hydrogel (GA-Gel) by disulfide bond reduction by GSH. Curcumin could be sustainedly released from the GA-gel through ester bond hydrolysis. Compared with curcumin and Nap-Cur, GA-Cur had more potent anti-cancer efficacy and higher cellular uptake for GA positive tumor cells *in vitro*. In conclusion, GA-Cur is a promising and potential therapeutic option for hepatocellular carcinoma therapy.

## Methods

### Solid phase peptide synthesis

Peptide derivatives of GA-GFFYKE-ss-ERGD and Nap-GFFYKE-ss-ERGD were synthesized by solid phase peptide synthesis (SPPS) using 2-chlorotrityl chloride resin and corresponding N-Fmoc protected amino acids with side chains properly protected by a tert-butyl group. The first amino acid (Fmoc-Asp(OtBu)-OH) was loaded to the resin at the C-terminal with the loading efficiency about 1.0 mmol/g. 20% piperidine in anhydrous N,N′-dimethylformamide (DMF) was used to remove Fmoc group. To couple the next Fmoc-protected amino acid, O(Benzotriazol-1-yl)-N,N,N′,N′-tetramethyluronium hexafluorophosphate (HBTU) was used as the coupling reagent. The peptide chain was grew according to the standard Fmoc SPPS protocol. At the final step, glycyrrhetinic acid (GA) or naphthalene acetic acid (Nap) was used. After the last coupling step, excessive reagents were removed through five times of DMF wash for 1 min, followed by five times of washing using dichloromethane (DCM) for 1 min. To cleave the peptide derivatives from the resin, ice-cold 95% TFA (2.5% of H_2_O and 2.5% of TIS) was used. The reaction solution was poured into ice-cold diethylether. The resulting precipitate was centrifuged for 10 min at 4 °C at a speed of 10,000 rpm. Afterward decanting the supernatant and the solid was dried by vacuum pump.

### Synthesis of Curcumin Glutaric acid (Cur-Gla)

Curcumin (1.107 g, 3 mmol) and Glutaric acid anhydride (0.353 g, 3.1 mmol) were dissolved in pyridine (23 mL), and the resulting solution was stirred at room temperature for 7 h. The solution was removed and the crude product was re-dissolved in ethylacetate (100 mL), which was washed with 1 M HCl (30 mL) to remove pyridine. This process was repeated for three times. The ethyl acetate was removed under vacuum to get the crude product. The product was purified via silica gel column chromatography, eluted with DCM: methanol (99:1, v/v) (yield: 49.2%).

### Synthesis the pro-gelator of GA-Cur and Nap-Cur

0.15 mmol of peptide derivative, GA-GFFYKE-ss-ERGD or Nap-GFFYKE-ss-ERGD was reacted with 48.3 mg of Curcumin Glutaric acid N-Hydroxysuccinimide (NHS) active ester (Cur-NHS) (0.1 mmol) inthe solvent of 3 mL of DMF containing 41.25 μL of diisopropylethylamine (DIPEA, 0.25 mmol). The resulting reaction mixture was stirred at room temperature overnight. The pro-gelators were obtained by high performance liquid chromatography (HPLC) with yields of 18–25%.

### Hydrogel formation

3 mg of GA-Cur or Nap-Cur was dissolved in 0.25 mL phosphate buffer saline (PBS) buffer solution (pH = 7.4, adjusted by 1.5 equiv. of Na_2_CO_3_). 4equiv. of glutathione (GSH) in 0.05 mL of PBS buffer (pH = 7.4, adjusted by 3.6equiv. of Na_2_CO_3_) was added to the above solution. Gels would form after being kept at room temperature (20–25 °C) for about 1.5 hours.

### Rheology Test

The rheology test was carried out on an AR 2000ex (TAInstrument) system. 25 mm parallel plates were used during the experiments at the gap of 500 μm. The solution of GA-Cur or Nap-Cur (1 wt%) with 4 equiv. of GSH was directly transferred to the rheometer and waited for 2 hours for the formation of gels. The dynamic strain sweep was performed at the frequency of 1 rad/s−1. A dynamic frequency sweep at the strain of 1% was then performed.

### Transmission electron microscopy (TEM)

15 μL of gel was placed on a carbon-coated copper grid and incubated for 60 seconds to allow the fibers to adhere to the copper grid. The sample was then rinsed thrice with ultrapure water. The sample was then stained with a saturated uranyl acetate solution and placed in a desiccator overnight prior to analysis.

### Release profile

A hydrogel in PBS (pH = 7.4) solution containing 1.0 wt% of pro-gelator was formed in an Eppendorf tube at 25 °C. After the gel was stable for 24 hours at 37 °C, 0.25 mL of PBS buffer solution was added on top of gels. 0.2 mL solution was taken out at the desired time point and 0.2 mL of fresh PBS was added back. We then monitored and calculated the release profile of Cur from the gel by a LCMS-20AD (Shimadzu) system. The experiment was performed at 37 °C and the results were calculated from three parallel experiments.

### Cell inhibition assay

The IC_50_ values of Cur, GA, GA-Cur, Nap-Cur, GA-gel, Nap-gel, GA + GA-Cur, GA + Nap-Cur were evaluated by the MTT assay. The HepG2 cells were seeded in 96-well plates at a density of 7,000 cells per well with a total medium volume of100 μL and then incubated for 24 hours. After removing the media, 100 μL of the media solutions containing a serial of concentrations of compound were added to the cells. 48 hours later, we replaced the medium with fresh medium supplemented with 5 μL MTT reagent (5 mg/mL). After another 4 hours, the medium containing MTT was removed and DMSO (100 μL/well) was added to dissolve the formazan crystals. A microplate reader (Bio-RAD iMarkTM, America) was used to measure the optical density of the solution at 490 nm. Cells without any treatments were used as the control. The inhibition capacity of GA + GA-Cur or GA + Nap-Cur was evaluated against HepG2 cells by pre-treating cells with 2 equiv. of free GA to block the GA receptors for 2 hours and then replacing with GA-Cur for another 48 hours. The same experiments were performed with 3T3 cells.

### Confocal microscopy

After being incubated for 24 h in 24-well plates at a density of 30,000 cells per-well, HepG2 cells were treated with 1 mL of DMEM solution containing 30 μM of different compounds for 4 h. The medium was then removed and the cells were washed for three times with fresh PBS. The images were recorded under the same detected conditions (excitation wavelength = 488 nm). The samples were then dyed with DAPI for 3 min. The experiments were carried out by using a laser scanning confocal microscope. The same experiments were performed with 3T3 cells.

### Cellular uptake

After being incubated for 24 h in 6-well plates at a density of 2.5 × 10^5^ cells per-well, HepG2 cells were treated with 2 mL growth medium containing 25 μM of Cur, GA-Cur, Nap-Cur, or GA + GA-Cur, respectively. In GA + GA-Cur group, 50 μM of GA was used to pre-treat cells for 2 hours. HepG2 cells were then rinsed for three times with PBS following with a treatment with 25 μM of GA-Cur for another 4 hours. After 4 hours’ incubation, cells were washed for three times with PBS to remove excess compounds and 500 μL of DMSO was added to each well to dissolve compounds in cells. The solution were collected after being treated with sonication for 15 min. The amount of compounds in the cells was determined by a microplate reader excitated at 488 nm. The same experiments were performed with 3T3 cells.

## Additional Information

**How to cite this article**: Chen, G. *et al*. A Glycyrrhetinic Acid-Modified Curcumin Supramolecular Hydrogel for liver tumor targeting therapy. *Sci. Rep.* **7**, 44210; doi: 10.1038/srep44210 (2017).

**Publisher's note:** Springer Nature remains neutral with regard to jurisdictional claims in published maps and institutional affiliations.

## Supplementary Material

Supplementary Information
