# Supplementary material for: A Glycyrrhetinic Acid-Modified Curcumin Supramolecular Hydrogel for liver tumor targeting therapy
Source: Sci Rep. 2017 Mar 10;7:44210. doi: 10.1038/srep44210 (PMC5345068; doi:10.1038/srep44210)
Supplement: Supplementary Information [file srep44210-s1.pdf]

Supporting Information

A Glycyrrhetinic Acid-Modified Curcumin Supramolecular Hydrogel for liver tumor targeting therapy

Guoqin Chen[1], Jinliang Li[2], Yanbin Cai[3], Jie Zhan[3], Jie Gao[4], Mingcai Song[1], Yang Shi[3] & Zhimou Yang[3]

Materials:

Curcumin was purchased from Aladdin. Glutaricanhydride was obtained from Alfa Aesar.18α-glycyrrhetinic acid was purchased from San bang. 2-Cl-trityl chloride resin (1.0–1.2 mmol/g) was obtained from Nankai University. O-(Benzotriazol-1-yl)-N,N,N',N'–tetramethyluroniumhexafluorophosphate (HBTU) and Fmoc-amino acids were obtained from GL Biochem (Shanghai). Dulbecco's modified Eagle's medium (DMEM), fetal bovine serum (FBS) and penicillin/streptomycin were purchased from Gibco Corporation. 3-(4, 5-Dimethylthiazol-2-yl)-2,5-diphenyl tetrazolium bromide (MTT) was purchased from Invitrogen (Grand Island, NY). Chemical reagents and solvents were used as received from commercial sources. Commercially available reagents and solvents were used without further purification, unless noted otherwise.

General methods:

^{1}H NMR (Bruker ARX 400) was used to characterize the synthesized compounds. Drug release was carried out by a LCMS-20AD (Shimadzu) system. TEM was performed at the Tecnai G2 F20 system, operating at 100 kV. Rheology test was done on an AR 2000 ex (TA instrument) system, 40 mm parallel plates was used during the experiment at the gap of 500 μm.

Peptide Synthesis

Peptide derivates ofGA-GFFYKE-ss-ERGD and Nap-GFFYKE-ss-ERGD were synthesiszed by solid phase peptide synthesis (SPPS) using 2-chlorotrityl chloride resin and corresponding N-Fmoc protected amino acids with side chains properly protected by a tert-butyl group. The first amino acid (Fmoc-Asp(OtBu)-OH) was loaded on the resin at the C-terminal with the loading efficiency about 1.4 mmol/g. 20% piperidine in anhydrous N,N'-dimethylformamide (DMF) was used to remove Fmoc group. Then to couple the next Fmoc-protected amino acid to the free amino group,O(Benzotriazol-1-yl)-N,N,N',N'-tetramethyluronium-hexafluorophosphate (HBTU) was used as

the coupling reagent. The peptide chain was grew according to the standard Fmoc SPPS protocol. At the final step, glycyrrhetinic acid and naphthalene acetic Acid was used to couplewith each of the peptide respectively. After the last coupling step, excessive reagents were removed through five times of DMF wash for 1 min, followed by five times of washing using dichloromethane (DCM) for 1 min. To cleave the peptide derivatives from the resin, ice-cold 95% TFA was used and the mixture was stirred, filtered at room temperature, and finally poured into ice-cold diethylether, successively. The resulting precipitate was centrifuged for 10 min at 3℃ at a speed of 10,000 rpm. Afterward decanting the supernatant and the solid was dried by vacuum pump.

Synthesis of Curcumin Glutaric acid (Cur-Gla)

Preparation of Cur-Gla: Curcumin (1.107 g, 3 mmol) and Glutaric anhydride (0.353 g, 3.1 mmol) were dissolved in pyridine (23 mL), and the resulting solution was stirred at room temperature for 7 h. The solution was then removed and the crude product was re-dissolved in ethylacetate (100 mL), and washed with 1 M HCl (30 mL) to remove pyridine.This process was repeated for three times. The ethyl acetate was removed under vacuum to get the crude product. The product was purified via silica gel column chromatography, eluted with DCM: Methanol (99:1, v/v) (yield 49.2%).

Synthesis the pro-gelator of GA-Cur and Nap-Cur

0.15 mmol of each of Peptide derivatives of GA-GFFYKE-ss-ERGD and Nap-GFFYKE-ss-ERGD was reacted with 48.3 mg of Curcumin Glutaric acid N-Hydroxysuccinimide (NHS) active ester (Cur-NHS) (0.1 mmol), respectively, in the solvent of 3 mL of DMF with 41.25 μL of diisopropylethylamine (DIPEA, 0.25 mmol). The resulting reaction mixture was stirred at room temperature overnight. The pro-gelators were obtained by HPLC (yields of 18-25%).

Characterization of GA-Cur:

^{1}H NMR (400MHz, DMSO-d_6) δ 7.51-7.61 (m,2H), 7.05-7.28 (m, 13H), 6.99-7.04 (d, 2H), 6.73-6.96 (m, 5H), 6.61-6.65 (s, 1H), 6.06-6.14 (s, 1H), 4.42-4.58 (m, 4H), 4.14-4.29 (m,4H), 3.75-3.90 (d, 6H), 3.24-3.32 (m, 3H), 2.93-3.10 (m, 6H), 2.70-2.75 (d, 4H), 2.60-2.67 (m, 2H), 2.14-2.36 (m, 10H), 1.67-1.91 (m, 9H), 1.42-1.64 (m, 9H), 1.10-1.33 (m, 12H), 0.86-1.03 (t, 11H), 0.60-0.74 (s, 6H). HR-MS: calc. M^+= 2398.7837, obsvd. 1/2$(M+H)^+$ = 1200.0502.

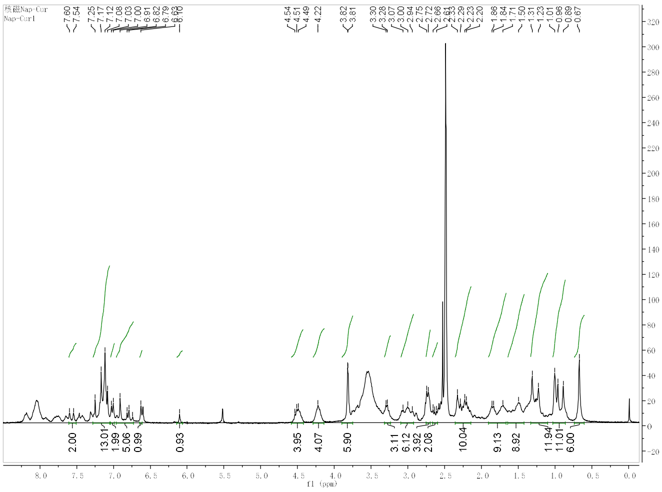

Fig. S-1. ^{1}H NMR of GA-Cur.

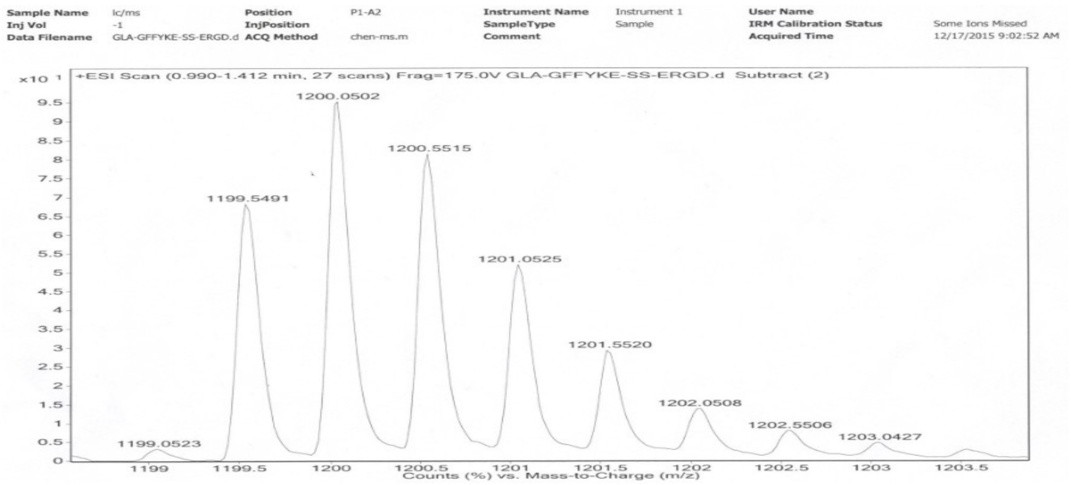

Fig. S-2. HR-MS of GA-Cur.

Nap-cur:

^{1}H NMR (400MHz, DMSO-d_6) δ 7.94-8.14 (m, 6H), 7.51-7.84 (m, 6H), 7.28-7.48 (m, 5H), 7.09-7.18 (m, 7H), 6.88-7.05 (m, 4H), 6.78-6.82 (m, 1H), 6.60-6.65 (d, 1H), 4.44-4.53 (m, 2H), 4.11-4.32 (m, 4H), 3.80-3.85(d, 4H), 3.24-3.33 (m, 2H), 2.87-3.12 (m, 6H), 2.69-2.77 (m, 4H), 2.54-2.62 (m, 2H), 2.31-2.36 (m, 2H), 2.15-2.27 (m, 4H), 1.66-1.92 (m, 6H), 1.35-1.53 (m, 4H), 1.20-1.27 (m, 2H). HR-MS: calc. M^+= 2114.3065, obsvd. 1⁄2$(M+H)^+$ = 1057.9146.

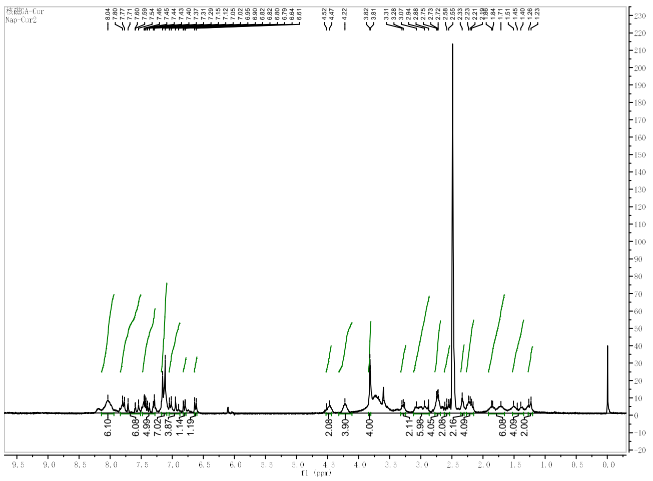

Fig. S-3. ^{1}H NMR of Nap-Cur.

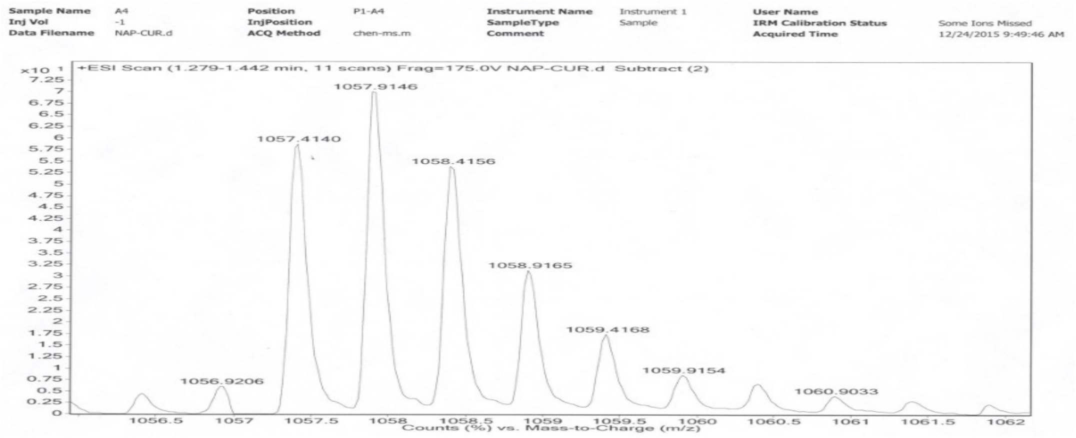

HR-MS of Nap-Cur(mw=2114.3065)

Hydrogel formation

3 mg of GA-Cur and Nap-Cur was dissolved in 0.25 mL PBS buffer solution (pH = 7.4, adjusted by 1.5 equiv. of Na_2CO_3), respectively. 4 equiv. of GSH in 0.05 mL of PBS buffer (pH = 7.4, adjusted by 3.6 equiv. of Na_2CO_3) was then added to the above solution. Gels would formafter being kept at room temperature 25 °C after about 1.5 hours.

Rheology

The rheology test was carried out on an AR 2000ex (TAInstrument) system, 25 mm parallel plates was used duringthe experiment at the gap of 500 μm. The solution of GA-gel upon adding 4 equiv. ofGSH was directly transferred to the rheometer and waited for 2 hours after the formation of gels, the

dynamic strain sweep wasperformed in the regionof 0.1–100 rad·s^{-1} at the strain of 1%.A dynamic strain sweep at thefrequency of 1 rad·s^{-1} was conducted finally.

Transmission electron microscopy (TEM)

TEM samples (GA-gel containing 1wt% compounds) were prepared at 35 °C and 15 µL sampleswas placed on a carbon-coated copper grid and incubated for60 seconds to allow the fibers to adhere to the substrate, then rinsed thrice with ultrapure water. The sampleswas then stained with a saturated uranyl acetate solution and be placed in a desiccator overnight prior to analysis.

Release profile

A hydrogel in PBS (pH = 7.4) solution containing 1.0 wt% of compound was formed in an Eppendorf tube at 25 °C. After the gel was stable for 24 hours at 37℃, 0.25 mL of PBS buffer solution was added on top of gels. 0.2 mL solution was taken out at the desired time pointand 0.2 mL PBS was added back. For the following timepoints, 0.2 mL of PBS was taken out and 0.2 mL of PBS wasadded back at each point. We then monitored and calculatedthe release profile from the gel formed by a LCMS-20AD (Shimadzu) system. The experiment was performed at 37 °C.

Cell inhibition assay

The IC_{50} values of Cur, GA, GA-Cur, Nap-Cur, GA-gel, Nap-gel, GA+GA-Cur, GA+Nap-Cur were evaluated by the MTT assay. The HepG2 cells were seeded in 96-well plates at a density of 7,000 cells per well with a total medium volume of100 µL and incubated for 24 hours. Then removed the media and 100 µL of the solutions containing a serial of concentrations of five compounds (Cur、Gla-cur-progel、Nap-cur-progel、Gla-cur-gel and Nap-cur-gel)were added into the cells. 48hours later, we replaced the medium with fresh medium supplemented with 5 µL MTT reagent (5 mg/mL). After 4 hours, the medium containing MTT was removed and DMSO (100 µL/well) was added to dissolve the formazan crystals. Amicroplate reader (Bio-RADiMarkTM, America) was used to measure the optical density of the solution at 490 nm. Cells without any treatment were used as the control.

Confocal microscopy

After being incubated for 24 h in 24-well plates at a density of 30000 cells per well, HepG2 cells was

treated with 1mL of DMEM solution containing 30 μM of curcumin contained above five compounds. The medium was removed and washed three times with fresh PBS ahead of being recored. The images were recorded under the same detected conditions (excitation wavelength = 488 nm) after incubating for 4h. And then the samples were dyed with Dapi for 3 min. This part of the experiment was carried out by using a laser scanning confocal microscope.

Cellular uptake

After being incubated for 24h in 6-well plates at 25 × 10^4 cells per well, HepG2 cells were treated in 2 mL growth medium containing 25 μM of Cur, GA-Cur, Nap-Cur and GA + GA-Cur respectively. In GA + GA-Cur group, 50 μM of GA was pretreated for 2 hours and HepG2 cells were then rinsed three times with PBS and treated with 25 μM of GA-Cur for a further 4 hours as mentioned above. After 4 hours incubation, the cells were washed 3 times with PBS to remove excess compounds and 500μL of DMSO was added to eachwell to dissolve compounds in cells. The solutionswascollected after treated with sonication for 15 min. The amount of compoundsin the cells was determined by microplate reader excitated at 488 nm.

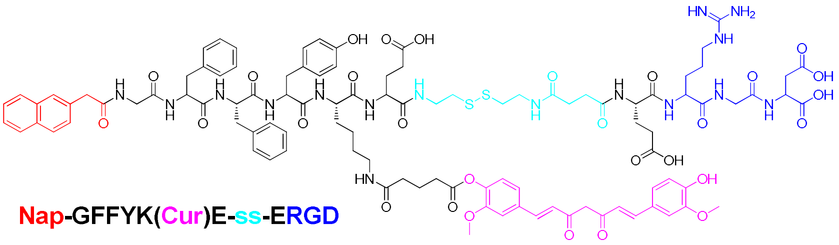

Fig. S-5. Chemical structures of Nap-Cur.

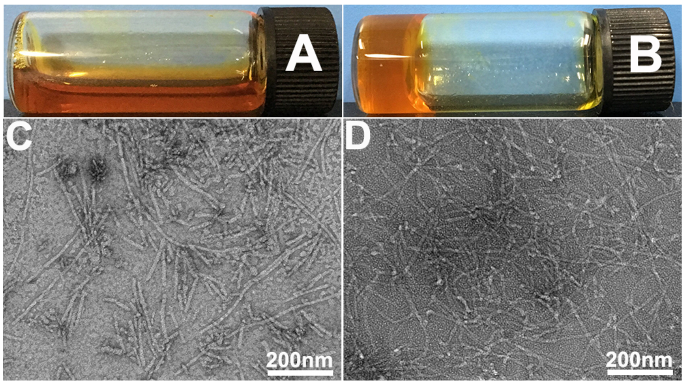

Fig. S-6. Optical images of A) PBS solutions containing 1 wt% of the precursors (Nap-Cur) and B) the hydrogel (Nap-gel) formed by treating solution in A) with 4 equiv. of GSH; transmission electron microscopy (TEM) image of A) the precursor (Nap-cur) and B) the hydrogel (Nap-gel).

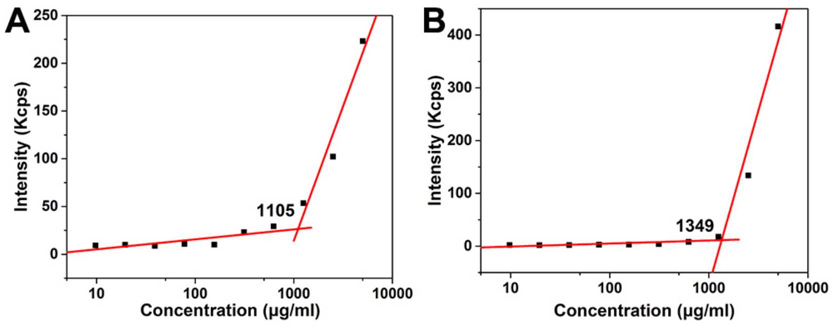

Fig. S-7. The critical micelle concentration (CMC) of A) GA-cur and B) Nap-cur.

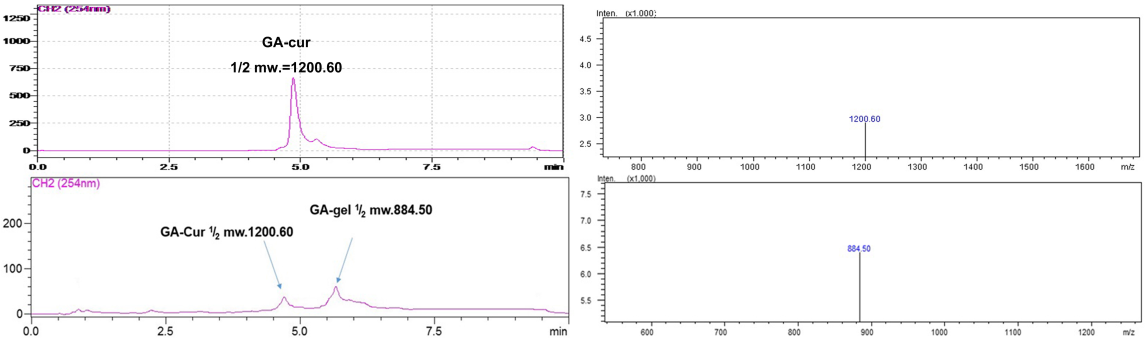

Fig. S-8. The LC-MS traces of the GA-Cur (mw=2398.78, $^{1}/_{2}$mw=1200.6) and the formed GA-gel(mw=1776.14, $^{1}/_{2}$mw=884.5).

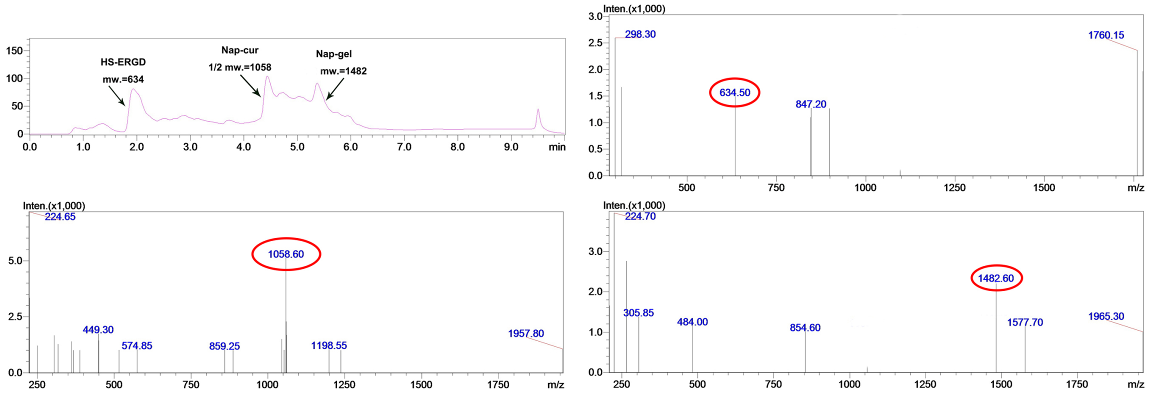

Fig. S-9. The LC-MS traces of the Nap-Cur (mw=2398.78, $^{1}/_{2}$mw=1058.6) and the formed Nap-gel(mw=1482.6)..

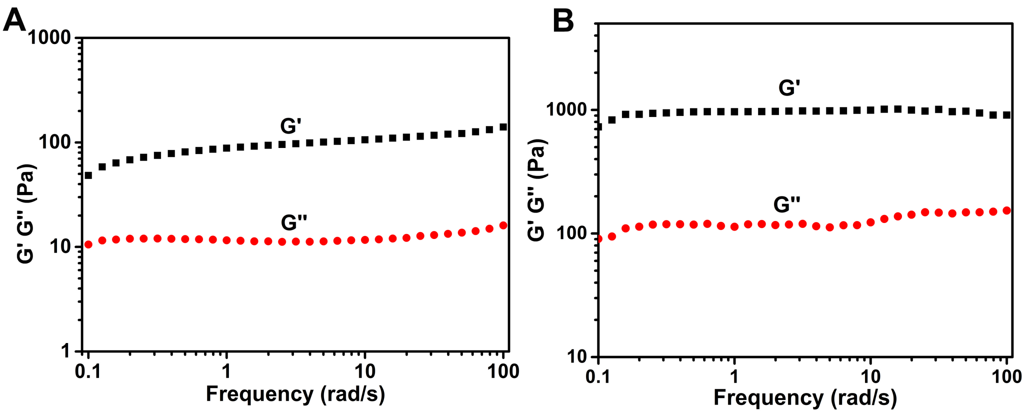

Fig. S-10. Dynamic frequency sweep of A) GA-gel and B) Nap-gel formed after 2 hours at the strain of 1% and at 25 °C.

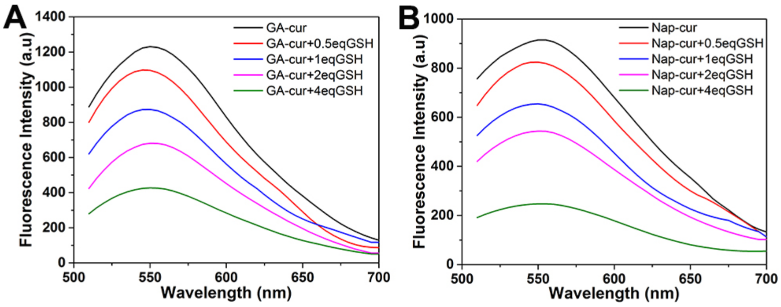

Fig. S-11. Fluorescent spectra of pro-gelators of A) GA-cur and B) Nap-cur with different amount of GSH at 25μM excited at 488nm (the results indicated the phenomenon of aggregation caused quenching).

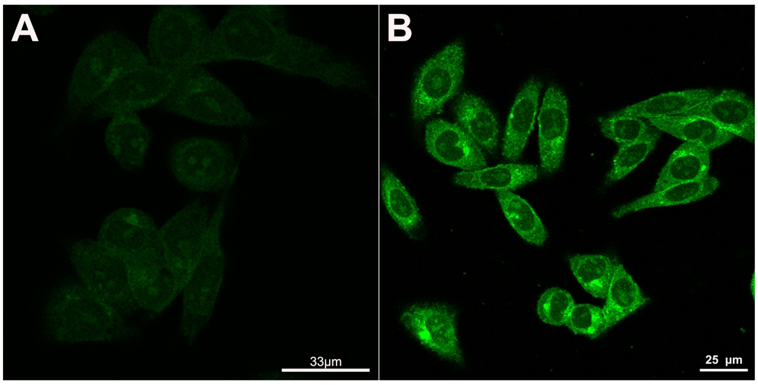

Fig. S-12. Confocal fluorescence microscopy images of HepG2 cells A) firstly treated with 2 equiv. GA for 2h and then with 25μM GA-cur for another 4h, B) treated with 2 equiv. GA and 25μM GA-cur for 4h simultaneously. (excitation wavelength = 488 nm)

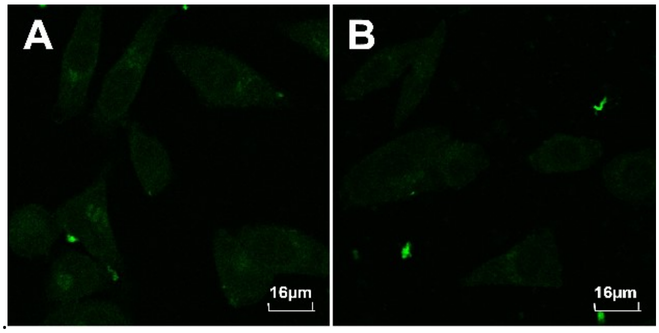

Fig. S-13. Confocal fluorescence microscopy images of HepG2 cells treated with A) GA-gel and B) Nap-gel at 4 h time point containing 25 µM curcumin (excitation wavelength = 488 nm)

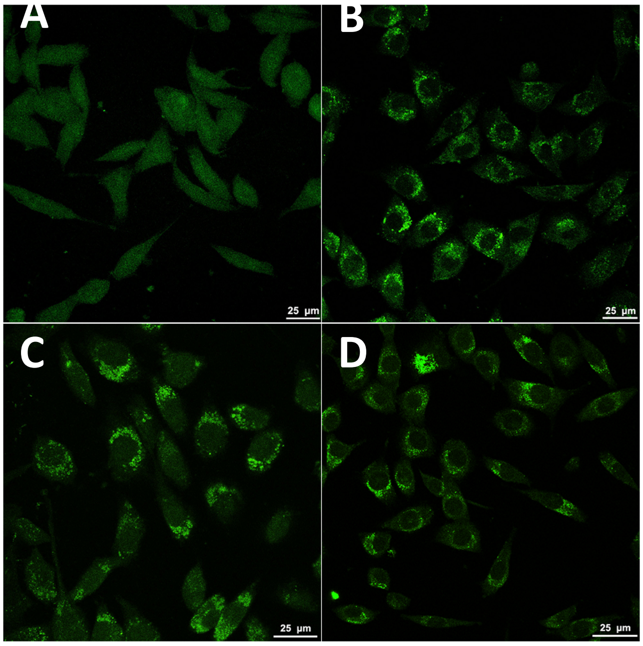

Fig. S-14. Confocal fluorescence microscopy images of 3T3 cells treated with 25 µM of A) Cur, B) Nap-Cur, C) GA-Cur, and D) GA+GA-Cur for 4 hours. (excitation wavelength = 488 nm).

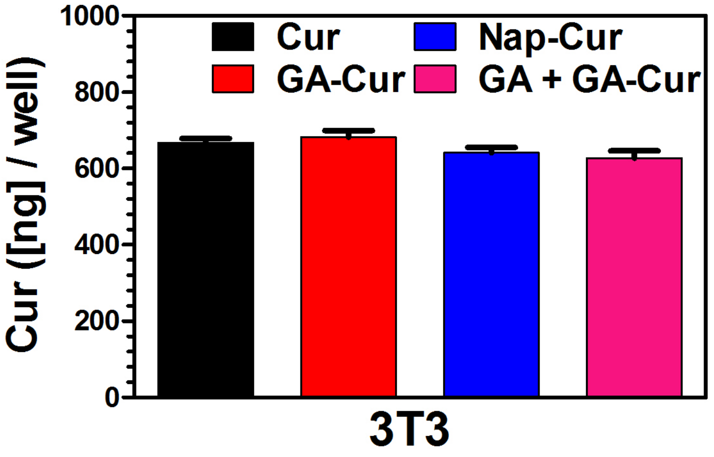

Fig. S-15. The amount of Curcumin in HepG2 cells treated with different compounds

	HepG2		NIH 3T3	
IC50 (μM)	mean	SD	mean	SD
Cur	26.48	1.46	28	2.72
GA-Cur	10.74	0.48	29.67	0.46
Nap-Cur	29.74	2.57	28.7	1.49
GA-gel	62.96	4.01	84.44	4.82
Nap-gel	77.86	3.55	75.07	3.10
GA + GA-Cur	28.02	2.45		

Table S-1. IC50 values (μM) of each compound for HepG2 cells and NIH 3T3 cells

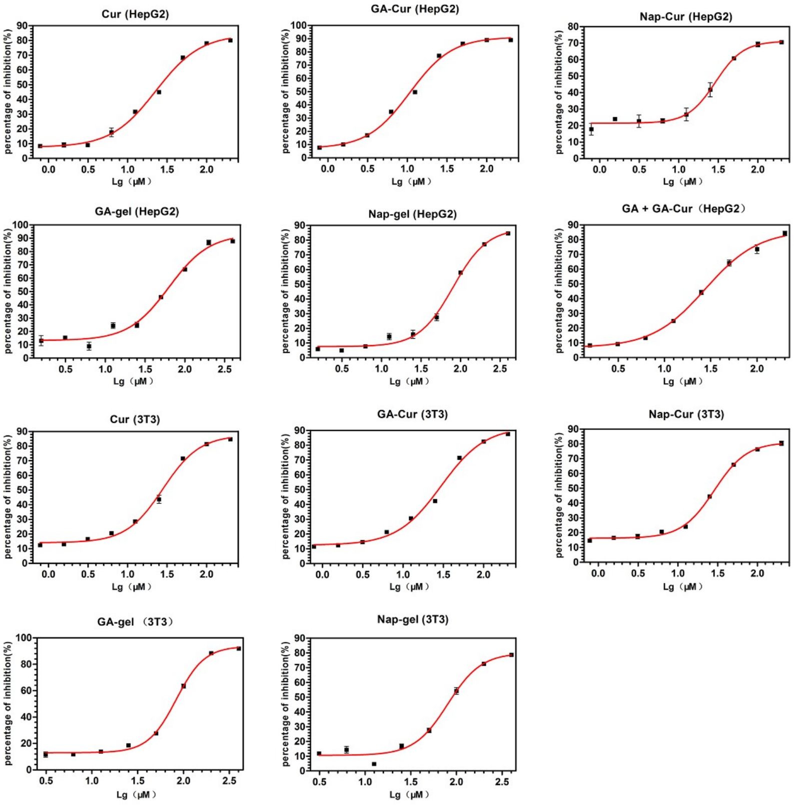

Fig. S-16. IC50 profile of each compound for HepG2 cells and NIH 3T3 cells
